# Minimally invasive approach for percutaneous CentriMag right ventricular assist device support using a single PROTEKDuo Cannula

**DOI:** 10.1186/s13019-016-0515-y

**Published:** 2016-08-04

**Authors:** Toshinobu Kazui, Phat L. Tran, Angela Echeverria, Catherine F. Jerman, Jessika Iwanski, Samuel S. Kim, Richard G. Smith, Zain I. Khalpey

**Affiliations:** 1 Department of Surgery, Banner University Medical Center, 1501 N. Campbell Avenue, Tucson, AZ 85724 USA; 2 Artificial Heart Program, Banner University Medical Center, 1501 N. Campbell Avenue, Tucson, AZ 85724 USA; 3 Department of Medical Pharmacology, University of Arizona College of Medicine, 1501 N. Campbell Avenue, PO Box 245017, Tucson, Arizona 85724 USA; 4 Department of Biomedical Engineering, University of Arizona College of Medicine, 1501 N. Campbell Avenue, PO Box 245017, Tucson, Arizona 85724 USA; 5 Department of Surgery, University of Arizona College of Medicine, 1501 N. Campbell Avenue, PO Box 245017, Tucson, Arizona 85724 USA; 6 College of Medicine, University of Arizona College of Medicine, 1501 N. Campbell Avenue, PO Box 245017, Tucson, Arizona 85724 USA

**Keywords:** Circulatory support, Heart failure, Minimally invasive surgery, Right ventricular assist device, PROTEKDuo

## Abstract

**Background:**

Right ventricular failure is a serious complication after left ventricular assist device placement.

**Case Presentation:**

A 70-year-old male in decompensated heart failure with right ventricular failure after the placement of a left ventricular assist device. A single dual-lumen PROTEKDuo cannula was inserted percutaneously via the internal jugular vein to draw blood from the right atrium and return into the pulmonary artery using the CentriMag system, by passing the failing ventricle. The patient was successfully weaned from right ventricular assist device.

**Conclusions:**

In comparison to two-cannula conventional procedures, this right ventrivular assist device system improves patient rehabilitation and minimizes blood loss and risk of infection, while shortening procedure time and improving clinical outcomes in right ventricular failure.

## Background

Perioperative right ventricular failure (RVF) is associated with a poor prognosis and high mortality [1]. After left ventricular assist device (LVAD) placement, 7–15 % of patients develop significant RVF that requires temporary right ventricular assist device (RVAD) placement [1, 2]. Temporary RVAD placement has previously required additional operations contributing to less satisfactory results compared to isolated continuous flow LVADs [3].

Traditionally, extracorporeal membrane oxygenation (ECMO) and RVAD insertion are established through sternotomy with direct right atrial and pulmonary artery (PA) cannulation or PA cannulation via graft. Other centers have proposed percutaneous RVAD placement through the right internal jugular vein (IJV) as well as femoral vein cannulation [4]. Impella RP (Abiomed, Inc, Danvers, MA, USA) is a temporary RVAD with percutaneous approach, however the access site is the femoral vein limiting patient ambulation and body position [5].

**Fig. 1:**
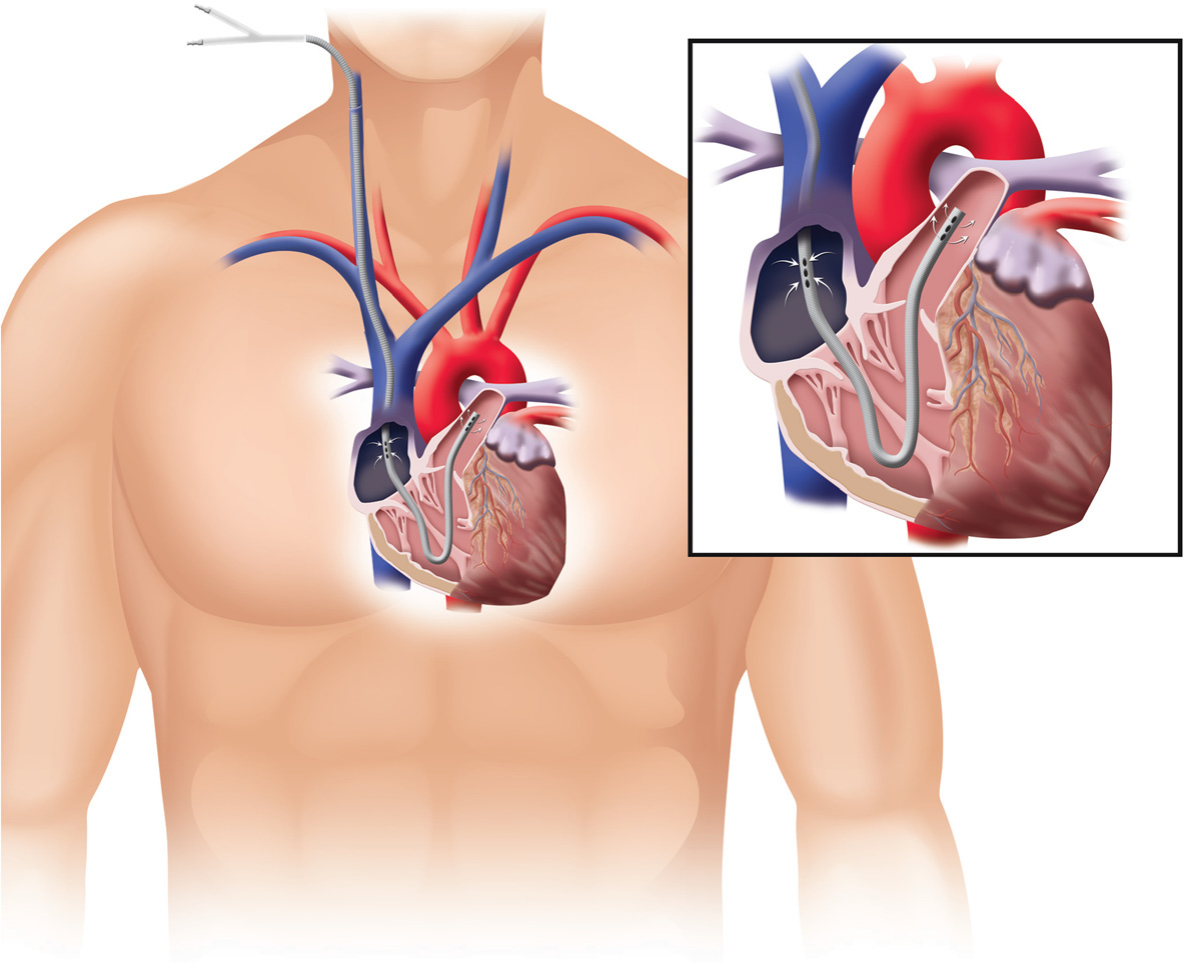
PROTEKDuo Cannula. The PROTEKDuo Cannula is a double lumen cannula. The proximal holes drain blood from right atrium to the pump. The distal holes return blood to the pulmonary artery

The dual-lumen PROTEKDuo (CadiacAssist, Inc. Pittsburgh, PA, USA) contains omnidirectional inflow and outflow ports for simultaneous drainage and reinfusion of blood [5]. Through a minimally-invasive approach via the IJV, the dual-lumen PROTEKDuo is positioned to draw blood from the right atrium and propel it into the PA, thereby bypassing the failing ventricle (Fig. 1).

This case describes successful restoration of RV function using a percutaneous single PROTEKDuo cannula through the right IJV with the CentriMag (Thoratec Corp, Pleasanton CA, USA) system and avoidance of re-sternotomy for decannulation.

## Case presentation

A 70-year-old male underwent Heartmate II (HMII) placement as a destination therapy. He had a history of atrial fibrillation, severe mitral stenosis, tricuspid regurgitation (TR), and non-ischemic cardiomyopathy (NICM). He was hospitalized with acute on chronic biventricular decompensated heart failure (EF 20–25 %) (NYHA IV, INTERMACS 2, ACC/AHA Stage D) four years after a mitral valve replacement and tricuspid valve repair. He had diabetes mellitus type 2, COPD, chronic kidney disease with baseline creatinine of 1.4–1.7 and on IV Lasix. He was suspected of multifocal pneumonia and an overall sequela of ongoing progressive advanced heart failure. Three weeks prior to LVAD implant, right heart cath showed RA of 10 mmHg, RV of 60/10 mmHg, PA of 60/28 (38) mmHg, PAWP of 22 mmHg, V-wave of 40 mmHg, Trans-Pulmonary Gradient of 16 mmHg, Thermodilution Cardiac Output of 4.85 L/min; Cardiac Index of 2.47 L/min/m^2^, Fick’s CO of 5.69 L/min and CI of 2.9 L/min/m^2^, PVR-Pulmonary Vascular Resistance of 3 Wood units, and SVR-Systemic Vascular Resistance of 627 dyn.s.cm^-5^. MRI Study showed (BSA-normalized values): RV EDV: 310 ml (156 ml normalized); RV ESV: 239 ml (120 ml normalized); RV SV: 72 ml (36 ml normalized); RV EF of 23 %); CCO of 5.8 L/min; CCI of 2.9 L/min/m^2^.

A re-do median sternotomy was performed and cardiopulmonary bypass established with bicaval cannulation. After a repeat of tricuspid value repair with Kay’s stitch due to severe regurgitation from annular dilatation, a HMII was inserted in standard fashion. The LVAD was initiated (4.5 L/min, 8200 rpms) but his RV function was acceptable; thus requiring inotropes support (DOB-5 mcg/kg/min; norepinephrine-15 mcg/kg/min, vasopressin-0.04 units/min and inhaled nitric oxide (iNO) of 40. Swan-Ganz catheter showed CVP of 27 mmHg, PA of 59/27(37) mmHg, CCO of 5.4 L/min, CCI of 2.6 L/min/m^2^, and SVR of 755.56 dyn.s.cm^-^5. TEE demonstrated descent RV function and he was extubated within 24-hours post-operation. Continuous renal replacement therapy (CRRT) was initiated on postoperative day (POD) two for his metabolic acidosis and anuria. The patient was re-intubated and sedated POD3 due to respiratory failure. He received one and two units of packed RBC on POD1 and POD3 respectively. No FFP, CRYO, or platelets were given. Epinephrine was started for RV support and LVAD flow decreased (5.0 L/min, 8400 rpms). Despite escalating vasopressor and inotropic support (Epi-2mcg/kg/min, DOB-5mcg/kg/min, Mil-0.125mcg/kg/min, norepinephrine-3mcg/kg/min, vaso-0.04units/min, Phenylephrine-75mcg/kg/min) echocardiography demonstrated refractory pulmonary hypertension and RVF with PVR of 3 Wood units. Swan-Ganz catheter showed CVP of 17 mmHg, PA of 42/22(29) mmHg, CCO of 5.8 L/min, CCI of 3.1 L/min/m^2^, SVR of 575 dyn.s.cm^-5^.

**Fig. 2:**
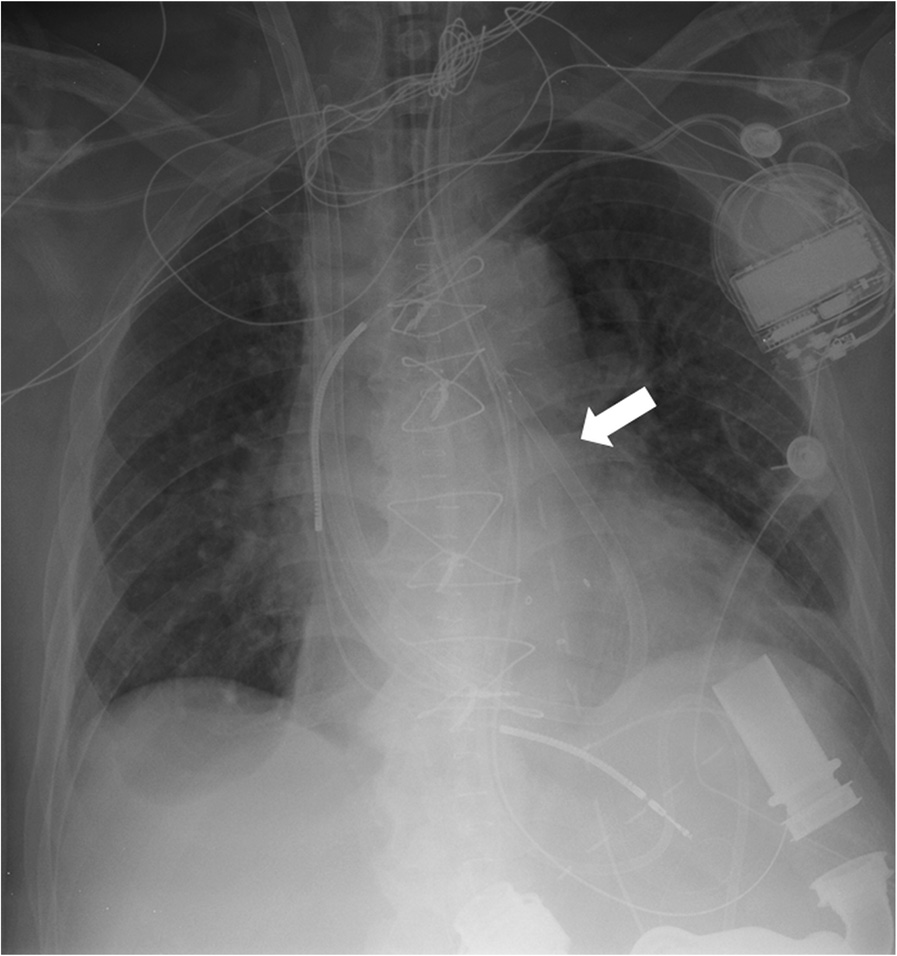
Placement of the PROTEKDuo Cannula. The PROTEKDuo cannula was placed from the right internal jugular vein extending through the right ventricle toward the pulmonary artery main trunk. The white arrow indicates the tip of cannula

A percutaneous CentriMag RVAD placement with a 29 Fr PROTEKDuo cannula was performed in the cardiac catheterization lab on POD6 (Fig. 2). Under fluoroscopy, a 0.025 T-J wire was passed through the Swan-Ganz catheter and subsequently exchanged for a stiffer 0.035 inch T-J wire, extending from the IJV, via brachiocephalic vein and superior vena cava, into the right atrium (RA) and ventricle, then through the pulmonary valve into the PA. The cannula was passed over the wire and positioned in the RA with outflow port beyond the pulmonary valve. The proximal and distal cannulae were then connected to the CentriMag system and RVAD was established with stable flow (3.5 L/min, 3800 rpms). LVAD flow was also increased (7 L/min, 8800 rpms). Cardiac output and hemodynamics improved exponentially after RVAD placement; reducing the need for pharmacologic support. Sildenafil and iNO were continued. Stabilized RV function overcame the patient’s pulmonary hypertension, reestablishing adequate pulmonary circulation and sufficient LV filling thereby enabling proper LVAD function and increased cardiac output. RVAD supported RV recovery facilitating the removal of 13 liters of fluid via CRRT, improving patient’s volume status. The patient’s heart was stable before device wean and decannulation took place on POD11.

## Conclusion

High mortality and a poor prognosis with RVF after LVAD implant indicate that treatment of RVF is an important factor in recovery from HF [6]. The approach described herein further improves upon previously documented minimally invasive procedures [4]. The PROTEKDuo was positioned via the IJV with inflow port in the RA and outflow port in the PA. Using the CentriMag system, RVAD was established to augment the failing ventricle. RV function was stabilized, restoring hemodynamics, overcoming the patient’s pulmonary hypertension, and allowing for 13 liters of fluid removal via CRRT. With improved RV function, the device was weaned and the patient decannulated eleven days later.

Conventional RVAD insertion often requires repeat sternotomy. In comparison, the percutaneous approach decreases blood loss and potential coagulopathy. Furthermore, the RV can be decompressed without chest opening. However, sometimes the chest is left open for coagulopathy and RV dilation, potentially increasing the risk of infection [7]. Surgical implantation has effectively facilitated longer support times than percutaneous RVADs, though utilizing femoral cannulation. Other percutaneous RVAD procedures involve separate inflow and outflow sites, generally including femoral cannulation [5]. The use of the dual-lumen PROTEKDuo requires only a single insertion site in the right jugular vein, which allows for expedited mobility and rehabilitation and minimizes lower limb ischemia. The cannula is a 510 K clearance which stated “a single cannula for both venous drainage and reinfusion of blood via internal jugular vein during extracorporeal life support (ECLS) procedure”. This 510 K approval process equals this product to the Avalon cannula made by Maquet. To our best knowledge, there is no FDA approval of ECLS comprising of pump, oxygenator, and cannula. Therefore, the use of PROTEKDuo Cannula with CentriMag pump is appropriately “off-label”. We cautioned that the maker of these cannula has the right not to sell the cannula. Other centers have proposed an RVAD implantation technique that does not necessitate resternotomy at the time of RVAD removal, but the concern of bleeding and infection from the Dacron graft site remains [7].

There are many advantages to using this PROTEKDuo Cannula as an RVAD with the CentriMag. First, the CentriMag is a reliable temporary pump and this off-pump catherization procedure can be performed in a short timeframe (<1 hours), minimizing blood loss, transfusion and postoperative inflammation. Furthermore, avoiding a median sternotomy, preserves the sternum for a later chest re-entry or heart transplant. Patients can be extubated early, and can ambulate early post-operatively without any groin instrumentation or cannulation; an advantage to previously reported percutaneous RVAD insertion [8]. Furthermore, the combination of CentriMag and PROTEKDuo cannula would allow us the option of slicing in an Oxygenator should the patient require one. Also, the cannula can robustly decompress the right heart allowing ventricular recovery and decreased clot formation because of the constant flow through the right ventricle.

Traditional sternal re-entry under emergent conditions is treacherous and cumbersome. This procedure has great utility in patients with previous cardiac surgery. The procedure is technically easy to establish RVAD support, provides efficient support and decompression in cardiogenic shock in coagulopathic patients. The disadvantage of having only a single cannula size, limits patients with a larger BSA. Also, Insertion of the cannula can be tricky requiring fluoroscopy and stiff guidewire to properly tunnel the cannula to the PA. Finally, we utilize heparin for anticoagulation under guidance of Rotational Thromboelastometry (ROTEM).

In conclusion, percutaneous PROTEKDuo cannula insertion with CentriMag system has provided a novel approach to establish RVAD support in a timely and minimally invasive technique. This method avoids unnecessary blood transfusion and resternotomy. Further study is warranted to further evaluate superiority over conventional RVAD approach.

## References

[1] Kormos RL, Teuteberg JJ, Pagani FD, Russell SD, John R, Miller LW (2010). Right ventricular failure in patients with the heartmate II continuous-flow left ventricular assist device: Incidence, risk factors, and effect on outcomes. J Thorac Cardiov Surg.

[2] Takeda K, Takayama H, Colombo PC, Jorde UP, Yuzefpolskaya M, Fukuhara S (2015). Late right heart failure during support with continuous-flow left ventricular assist devices adversely affects post-transplant outcome. J Heart Lung Transplant.

[3] Lazar JF, Swartz MF, Schiralli MP, Schneider M, Pisula B, Hallinan W (2013). Survival after left ventricular assist device with and without temporary right ventricular support. Ann Thorac Surg.

[4] Takayama H, Naka Y, Kodali SK, Vincent JA, Addonizio LJ, Jorde UP (2012). A novel approach to percutaneous right-ventricular mechanical support. Eur J Cardiothorac Surg.

[5] Cheung AW, White CW, Davis MK, Freed DH (2014). Short-term mechanical circulatory support for recovery from acute right ventricular failure: Clinical outcomes. J Heart Lung Transplant.

[6] Takeda K, Naka Y, Yang JA, Uriel N, Colombo PC, Jorde UP (2014). Outcome of unplanned right ventricular assist device support for severe right heart failure after implantable left ventricular assist device insertion. J Heart Lung Transplant.

[7] Saeed D, Maxhera B, Kamiya H, Lichtenberg A, Albert A (2015). Alternative right ventricular assist device implantation technique for patients with perioperative right ventricular failure. J Thorac Cardiov Surg.

[8] Takayama H, Soni L, Kalesan B, Truby LK, Ota T, Cedola S (2014). Bridge-to-decision therapy with a continuous-flow external ventricular assist device in refractory cardiogenic shock of various causes. Circ Heart Fail.

